# Supplementary material for: NIR-II bioluminescence for in vivo high contrast imaging and in situ ATP-mediated metastases tracing
Source: Nat Commun. 2020 Aug 21;11:4192. doi: 10.1038/s41467-020-18051-1 (PMC7442788; doi:10.1038/s41467-020-18051-1)
Supplement: Supplementary file 1 — Supplementary Information [file 41467_2020_18051_MOESM1_ESM.pdf]

Supplementary Information

NIR-II Bioluminescence Probes for in Vivo High Contrast Imaging and in Situ ATP-Mediated Metastases Tracing

Lu *et.al.*

Supplementary Methods

Synthesis and characterizations of Cy5 and Cy7.5.

Cy5 and Cy7.5 are synthesized as illustrated in Supplementary Figure 1.

Synthesis of 1-ethyl-2,3,3-trimethyl-3H-indol-1-ium iodide (1).

2,3,3-trimethyl-3H-indole (1.59 g, 10 mmol) was dissolved in 5 mL of ethyl iodide and refluxed at 80 ^{o}C overnight. The reaction mixture was then cooled to room temperature and ether was used to precipitate the product from solution to yield 2.8 g (89%) of compound **1**.

Synthesis of 3-ethyl-1,1,2-trimethyl-1H-benzo[e]indol-3-ium iodide (2).

1,1,2-Trimethyl-1H-benz[e]indole (1.05 g, 5 mmol) was dissolved in 5 mL of ethyl iodide and refluxed at 80 ^{o}C overnight. The reaction mixture was then cooled to room temperature and ether was used to precipitate the product from solution to yield 1.5 g (82%) of compound **2**.

Synthesis of Cy5.

To a mixture of N-[3-(phenylamino)allylidene]aniline monohydrochloride (258.8 mg, 1 mmol), NaOAc (82 mg, 1 mmol) and Compound **1** (630.4 mg, 2 mmol) was added Ac_2O (8 mL) and subsequently heated at 100 ^{o}C under nitrogen for 2 h. After cooling to room temperature, ether was poured into the solution and the precipitate was purified by a flash column chromatography [silica gel, MeOH/DCM = 0/100 to 1/100, v/v] to give Cy5 (yield: 403 mg, 75%). 1H NMR (400 MHz, CDCl3) δ 8.03 (t, J = 13.0 Hz, 2H), 7.62 (d, J = 7.9 Hz, 2H), 7.28 (t, J = 7.8 Hz, 2H), 7.23 (t, 6.0 Hz, 2H), 7.08 (d, J = 8.0 Hz, 2H), 6.80 (t, J = 12.5 Hz, 1H), 6.31 (d, J = 13.6 Hz, 2H), 4.11 (q, J = 7.0 Hz, 4H), 1.73 (s, 12H), 1.41 (t, J = 7.2 Hz, 6H); Maldi-Tof/Tof-MS: calculated for C29H35N2+ [M]+, 411.2795; Found, 411.2344. [M]+.

Synthesis of Cy7.5.

To a mixture of N-[5-(phenylamino)-2,4-pentadienylidene]aniline monohydrochloride (284.78 mg, 1 mmol), NaOAc (82 mg, 1 mmol) and Compound **2** (730.5 mg, 2 mmol) was added Ac_2O (8 mL) and subsequently heated at 100 ^{o}C under nitrogen for 2 h. After cooling to room temperature, ether was poured into the solution and the precipitate was purified by a flash column chromatography [silica gel, MeOH/DCM = 0/50 to 1/50, v/v] to give Cy7.5 (yield: 400 mg, 60%). 1H NMR (400 MHz, CDCl3) δ 8.12 (d, J = 8.6 Hz, 2H), 7.94 (t, J = 16.2 Hz, 7H), 7.59 (t, J = 7.6 Hz, 2H), 7.44 (t, J = 7.5 Hz, 2H), 7.35 (d, J = 8.8 Hz, 2H), 6.69 (s, 2H), 6.28 (d, J = 12.1 Hz, 2H), 4.23 (d, J = 6.4 Hz, 4H), 1.99 (s, 12H), 1.48 (t, J = 7.1 Hz, 6H); Maldi-Tof/Tof-MS: calculated for C39H41N2+ [M]+, 537.3264; Found, 537.2692. [M]+.

Synthesis and characterizations of FD-1029.

FD-1029 is synthesized as illustrated in Supplementary Figure 2.

Synthesis of compound 1.

2-Methylindole (2.0 mmol, 262 mg) and 60% Sodium hydride (2.1 mmol, 84 mg) were mixed in anhydrous tetrahydrofuran (10 mL) and stirred at 0 ^{o}C for 20 min. To this solution was added butanesultone (2.0 mmol, 272 mg) and then heated at 100 ^{o}C for 2 h. After cooling, the solution was treated with isopropyl alcohol. The white solid was collected by filtration to afford compounds **1** (yield 95%).

Compound **1:** ^{1}H NMR (400 MHz, DMSO-d_6) δ 7.41-7.36 (m, 2H), 7.05-7.01 (m, 1H), 6.96-6.92 (m, 1H), 6.17 (s, 1H), 4.10-4.07 (t, 2H), 2.49-2.46 (m, 2H), 2.40 (s, 3H), 1.73-1.68 (m, 2H), 1.65-1.59 (m, 2H). ^{13}C NMR (101 MHz, DMSO-d_6) δ 136.9, 136.8, 128.0, 120.4, 119.5, 119.2, 109.8, 99.8, 51.4, 42.8, 29.5, 23.1, 12.9.

Synthesis of dyes FD-1029.

Compound **1** (4.3 mmol, 1.14 g) and acetyl chloride (2.2 mmol, 172 mg) in acetic anhydride (20 mL) were heated at 55 ^{o}C for 4h. N-[(3-(Anilinomethylene)-2-chloro-1-cyclohexen-1-yl)methylene]aniline monohydrochloride (2.2 mmol, 720 mg) was added to the mixed solution, and then heated at 100 ^{o}C for 1.5 h. After cooling, the solution was treated with ether (60 mL). The precipitate was collected by filtration. The product was purified by preparative RP HPLC (Waters C18 (5 μm, 250 mm × 20 mm) (yield 90%).

Compound **FD-1029.** ^{1}H NMR (400 MHz, DMSO-*d*$_6$) δ 7.65-7.60 (m, 6H), 7.30-7.19 (m, 8H), 7.07-7.05 (m, 6H), 4.28 (s, 8H), 2.87 (m, 4H), 2.49-2.45 (t, 8H), 2.23 (s, 12H), 1.93-1.91 (m, 2H), 1.79-1.77 (m, 8H), 1.70-1.64 (m, 8H). ^{13}C NMR (101 MHz, DMSO-*d*$_6$) δ 157.9, 152.3, 146.5, 144.4, 137.5, 132.4, 127.3, 126.4, 123.4, 122.5, 119.9, 119.2, 111.4, 51.2, 49.0, 43.8, 29.5, 28.9, 22.9, 12.7. HRMS (ESI) $[C_{64}H_{70}ClN_4O_{12}S_4]^{3-}$: calculated: 416.4526, measured: 416.4532.

Calculation of FRET efficiency and BRET ratio.

FRET efficiencies between organic dyes were calculated according to the equation:

$$E = 1 - F'_D/F_D \qquad (1)$$

where F'_D and F_D are the donor fluorescence intensities with and without an acceptor, respectively.[1] The optimal FRET efficiency between Cy5 and Cy7.5 was calculated to be 64.1% (Supplementary Figure 6A). The optimal FRET efficiency between Cy7.5 and FD-1029 was calculated to be 90.8% (Supplementary Figure 6B).

The Förster distances (R_0) of these two steps were also calculated to be1.3 nm and 6.4 nm according to the equations:

$$\mathrm{J}(\lambda) = [\int_0^\infty \mathrm{F_D}(\lambda)\,\varepsilon_\mathrm{A}(\lambda)\,\lambda^4\,\mathrm{d}\lambda]\,/[\int_0^\infty \mathrm{F_D}(\lambda)\mathrm{d}\lambda] \qquad (2)$$

$$R_0 = 0.0211[\kappa^2 n^{-4}\Phi_\mathrm{D}\mathrm{J}(\lambda)]^{1/6} \qquad (3)$$

where $F_D(\lambda)$ is the area-normalized emission spectrum of donor, $\varepsilon_A(\lambda)$ is the molar absorption spectrum of the acceptor in $M^{-1}cm^{-1}$, λ is the wavelength in nm, κ^2 is orientation factor (κ^2=2/3 due to dynamic averaging donor-acceptor systems), Φ_D is quantum yield of the donor, and *n*=1.35 is the refractive index of the surrounding medium.[2]

BRET ratio was defined by the acceptor emission relative to the donor emission.[3] As illustrated in Supplementary Figure 6F, area A is the integrated total emission (625-800 nm) from Cy5 and area B is the integrated total emission from luciferase (500-625 nm). Thus, the BRET ratio of luciferase-Cy5 is 4.3. Similarly, the BRET ratio of NIR-II-BPs was calculated to be 4.2 through dividing the intensity of acceptor emission (625-1400 nm) by that of the donor emission (500-625 nm).

Rose criterion.

The Rose criterion (named after Albert Rose) states that an SNR of at least 5 is needed to be able to distinguish image features with certainty. An SNR lower than 5 means less than 100% certainty in identifying image details. This is an important criterion used by physicians to judge object conspicuity based on SNR in clinical image analysis and can be adopted in our work for the discernible signal in imaging.

Supplementary Figures

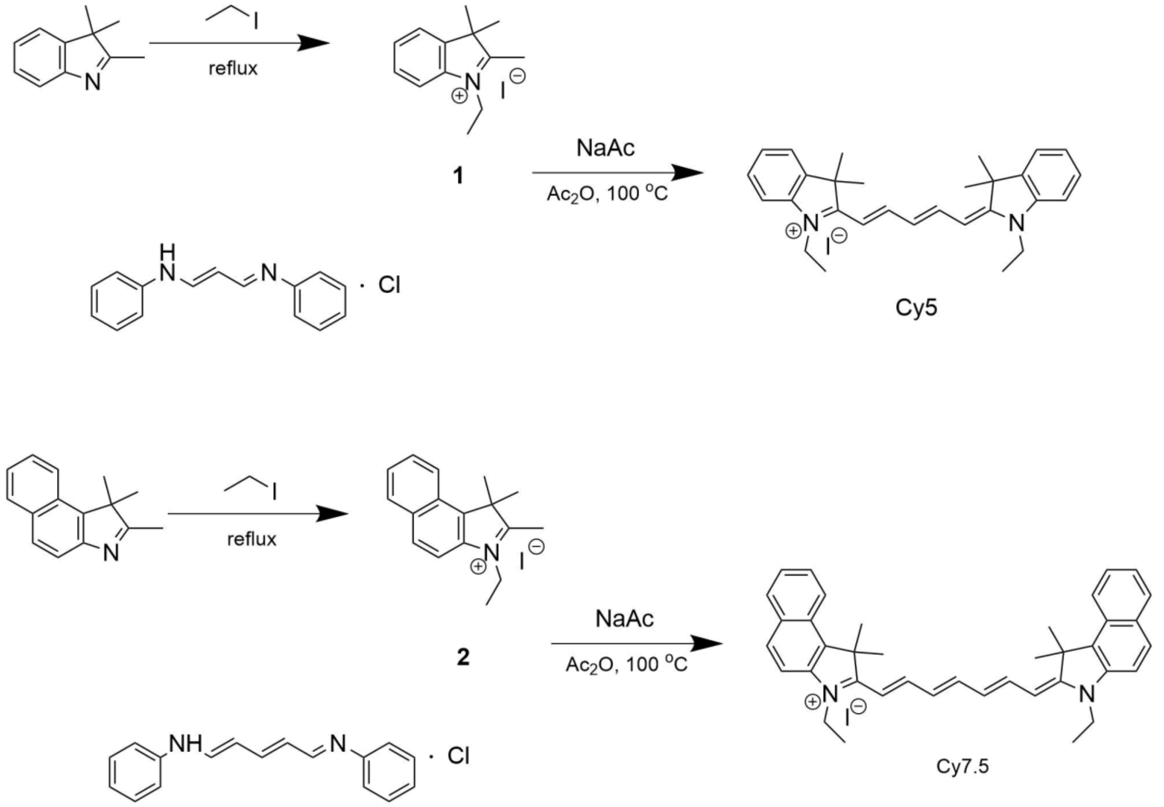

Supplementary Figure 1. Synthesis of compounds Cy5 and Cy7.5 dyes.

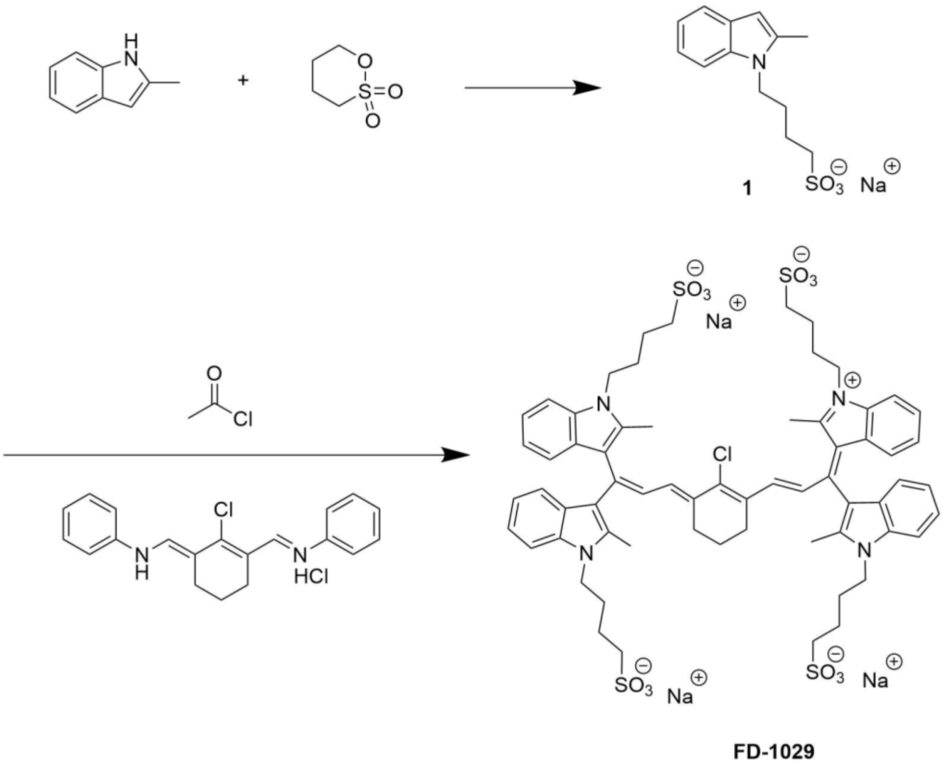

Supplementary Figure 2. Synthesis of compounds FD-1029 dyes.

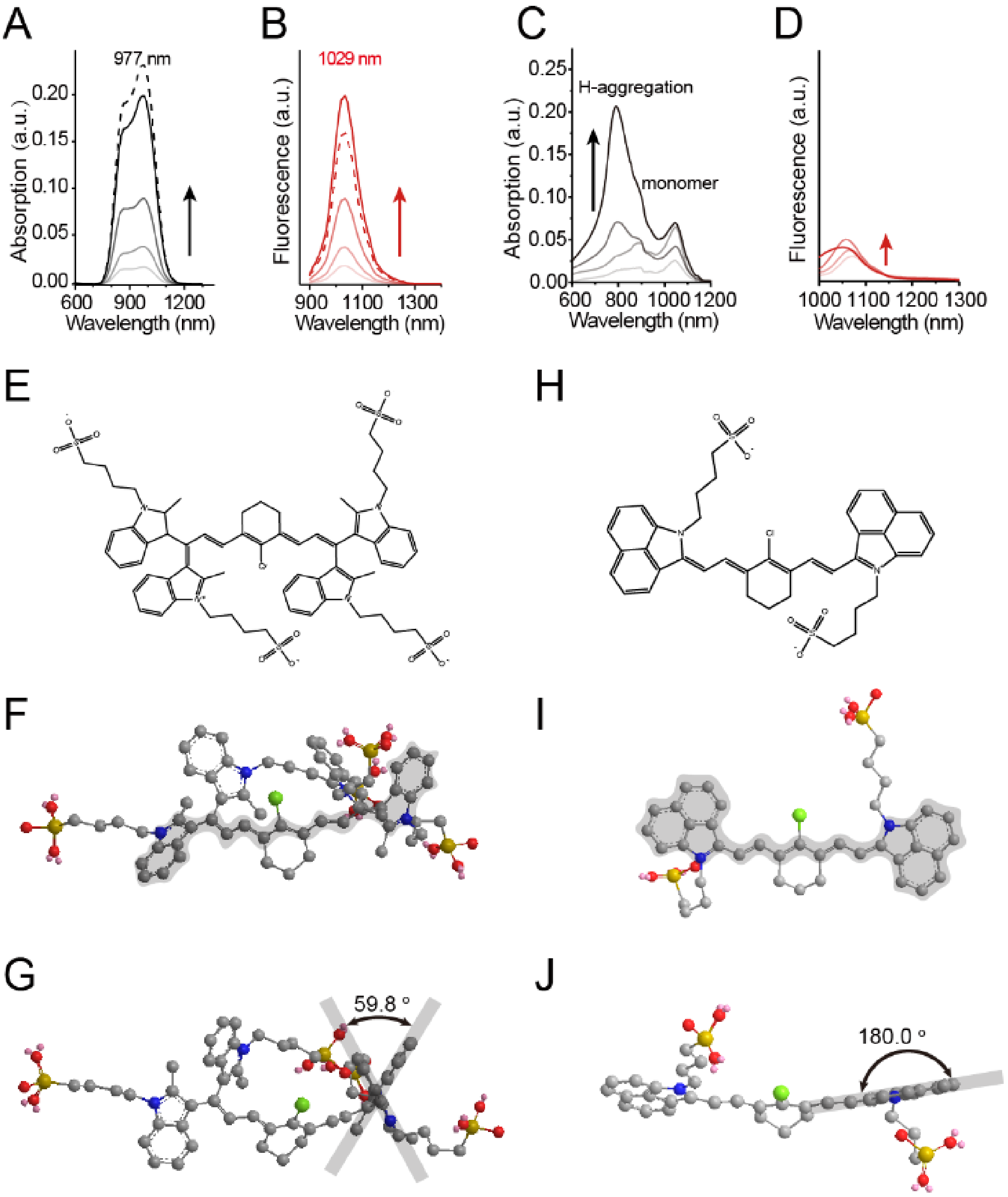

Supplementary Figure 3. (A) Absorbance spectra of FD-1029 in DSPE-PEG2000 micelles with the concentration of 1, 2, 5, 10 μM (from bottom to top). The dashed line refers to 12 μM FD-1029 in micelles. (B) Fluorescent emission spectra of FD-1029 in DSPE-PEG2000 micelles with the concentration of 1, 2, 5, 10 μM (from bottom to top). The dashed line refers to 12 μM FD-1029 in micelles. (C) Absorbance spectra of FD-1080 in DSPE-PEG2000 micelles with the concentration of 1, 2, 5, 10 μM (from bottom to top). (D) Fluorescent emission spectra of FD-1080 in DSPE-PEG2000 micelles with the concentration of 1, 2, 5, 10 μM (from bottom to top). (E) The chemical structure of FD-1029. (F, G) Ball-and-stick models of FD-1029. The shaded part shows the conjugated π-electron system (F) and the ipsilateral terminal groups (G) of FD-1029. (H) The chemical structure of FD-1080. (I, J) Ball-and-stick models of FD-1080, which were performed with ChemDraw3D 18.0. The shaded part shows the conjugated π-electron system (I) and the ipsilateral terminal groups (J) of FD-1080.[4, 5]

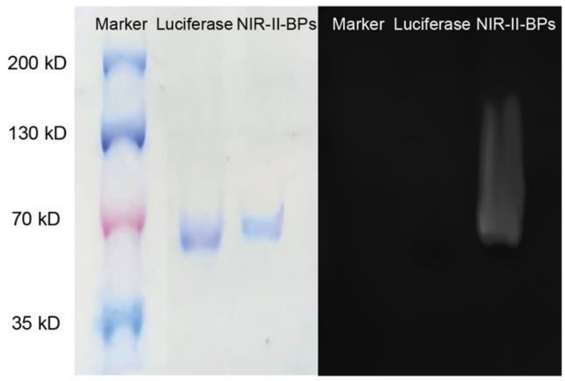

Supplementary Figure 4. SDS PAGE gel analysis of NIR-II-BPs in TBE-SDS buffer.[6] The optical photo (left) and the NIR-II imaging photo (right) of Marker (lane 1), luciferase (lane 2), and NIR-II-BPs (lane 3). Repeated for 3 times in independent experiments.

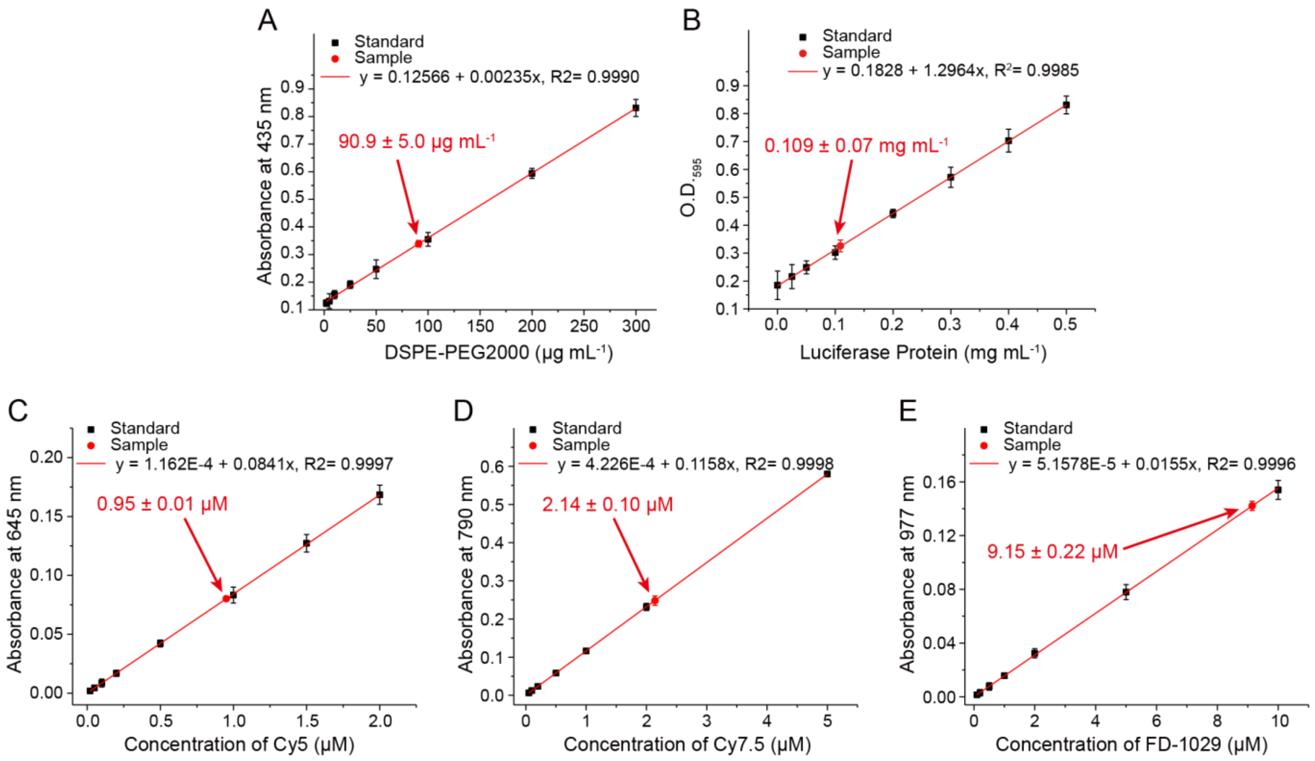

Supplementary Figure 5. (A) Determination of the DSPE-PEG2000 in NIR-II-BPs with a colorimetric method.[7] As the molar mass of DSPE-PEG2000 is 2805.5, molar concentration of DSPE-PEG2000 in NIR-II-BPs is 32.4 ± 1.8 nmol mL^{-1}. (B) Determination of the concentration of luciferase protein in NIR-II-BPs using the Bradford assay.[8] As the molar mass of luciferase is ~62 KDa, molar concentration of luciferase in NIR-II-BPs is 1.76 ± 0.12 nmol mL^{-1}. (C) The concentration of Cy5 in NIR-II-BPs was determined to be 0.95 ± 0.01 μM with spectrophotometric method. (D) The concentration of Cy7.5 in NIR-II-BPs was determined to be 2.14 ± 0.10 μM with spectrophotometric method. (E) The concentration of FD-1029 in NIR-II-BPs was determined to be 9.15 ± 0.22 μM with spectrophotometric method. Data points with error bars in **A-E** are presented as mean ± s.d. derived from n = 3 independent detection. Source data are provided as a Source Data file.

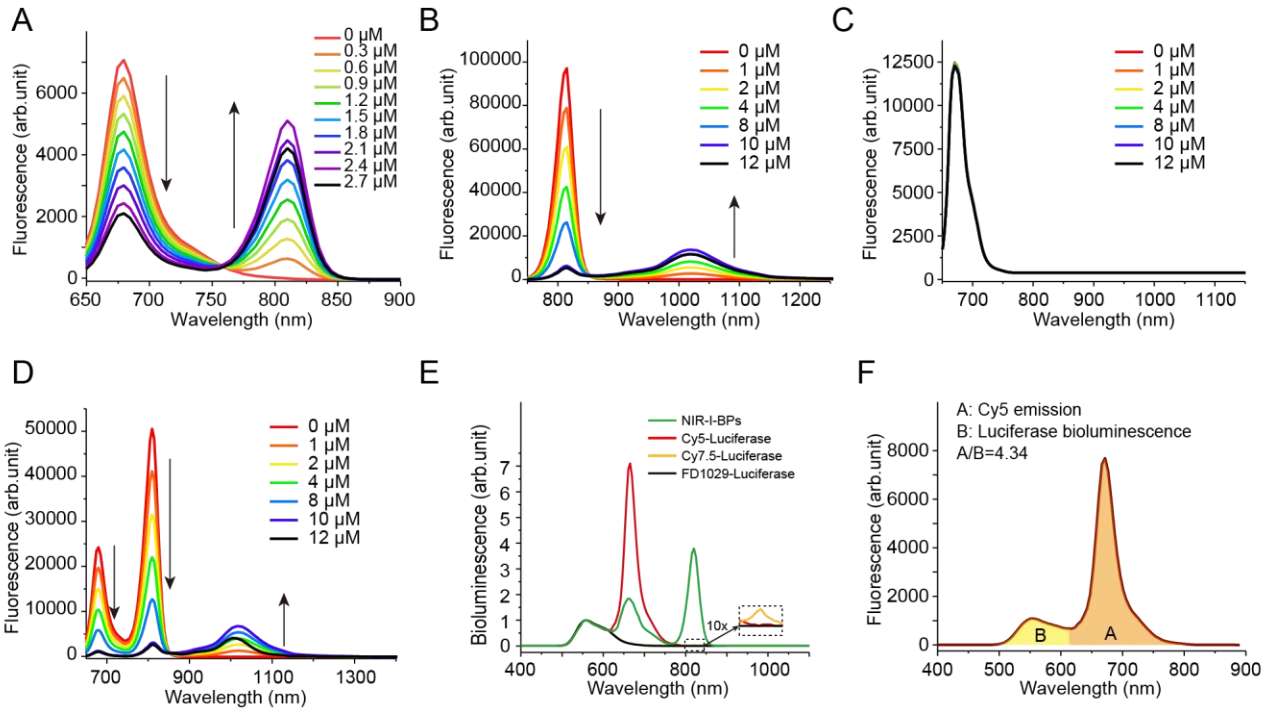

Supplementary Figure 6. (A) Fluorescence spectra of Cy5, Cy7.5 co-loaded micelles with 1 μM Cy5 and increasing concentration of Cy7.5. (B) Fluorescence spectra of Cy7.5 and FD-1029 co-loaded micelles with 2.4 μM Cy7.5, and increasing concentration of FD-1029. (C) Fluorescence spectra of Cy5, FD-1029 co-loaded micelles with 1 μM Cy5, and increasing concentration of FD-1029. (D) Fluorescence spectra of Cy5, Cy7.5, and FD-1029 co-loaded micelles with 1 μM Cy5, 2.4 μM Cy7.5, and increasing concentration of FD-1029. (E) Bioluminescence emission spectrum of different organic dyes loaded micelles-luciferase conjugates. The spectrum is normalized by the maximum bioluminescence intensity of luciferase at 560 nm. NIR-I-BPs refers to Cy5, Cy7.5 co-loaded micelles-luciferase conjugates. (F) Bioluminescence emission spectrum of Cy5 loaded micelles-luciferase conjugates.

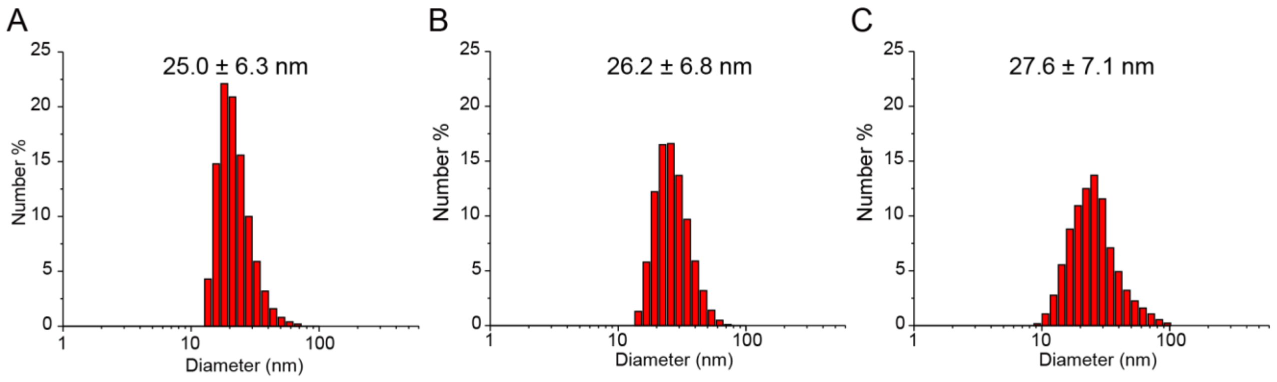

Supplementary Figure 7. Dynamic light scattering (DLS) measurement of NIR-II-BPs in PBS buffer and mouse serum. (A) As-made NIR-II-BPs. (B) NIR-II-BPs stored in PBS buffer for one week. (C) NIR-II-BPs stored in mouse serum for one week.

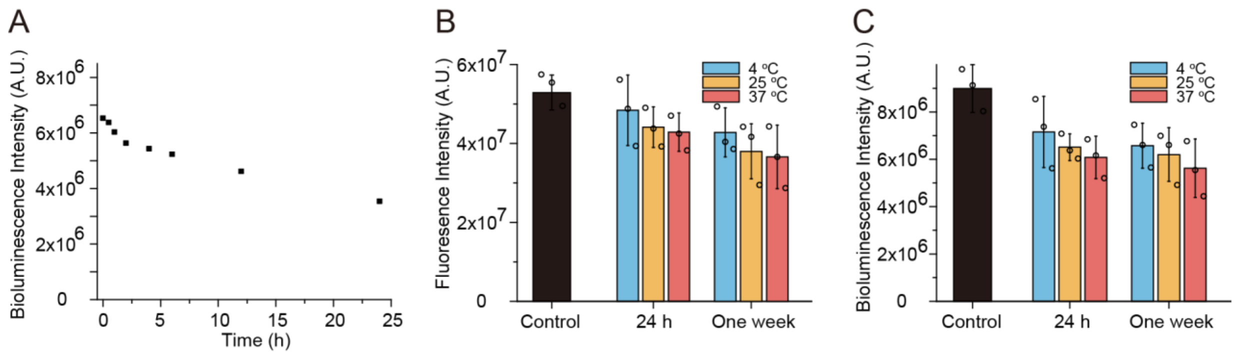

Supplementary Figure 8. Stability of NIR-II-BPs. (A) NIR-II bioluminescence intensity of NIR-II-BPs (10 μg mL^{-1}) in PBS (pH = 7.4) over 24 hours. (B) NIR-II fluorescence intensity of NIR-II-BPs in mouse serum after storing at varied temperatures for 24 hours and for one week. Bars are presented as mean ± s.d. derived from three separate measurements. (C) NIR-II bioluminescence intensity of NIR-II-BPs in mouse serum after storing at varied temperatures for 24 hours and for one week. Bars are presented as mean ± s.d. derived from three separate measurements. Source data are provided as a Source Data file.

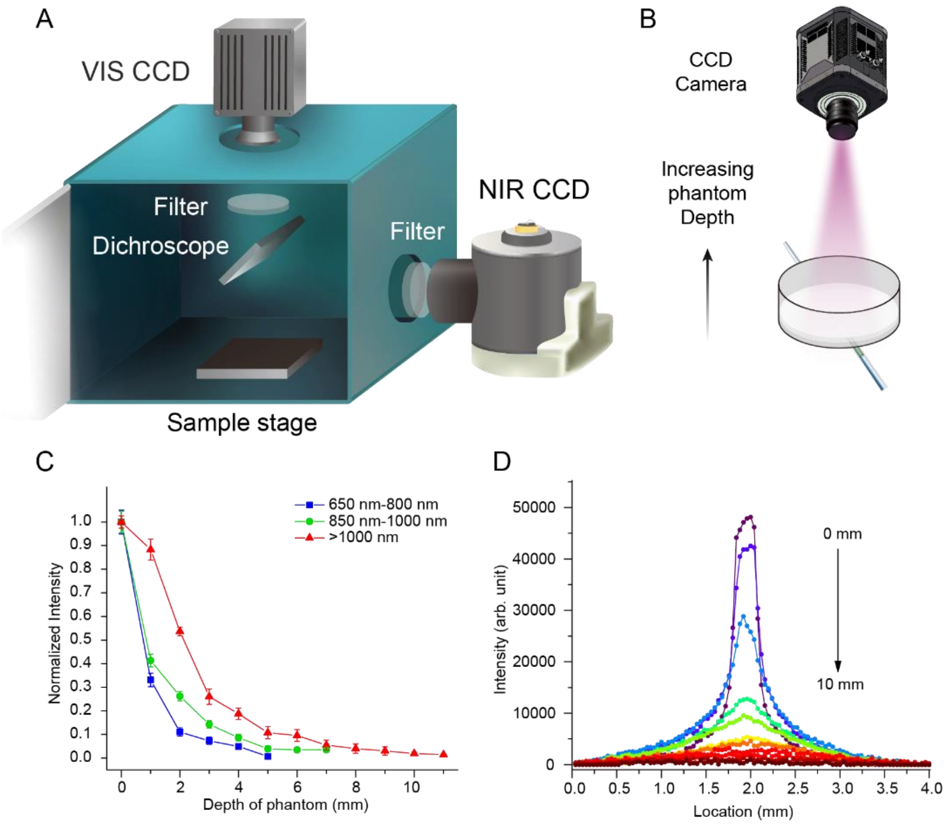

Supplementary Figure 9. (A) Illustration of VIS & NIR imaging system. (B) Scheme of bioluminescence imaging setup for tissue penetration detection. (C) Intensity decay of the NIR-II-BPs filled capillary tube as a function of depth in Intralipid in different wavelengths sub-regions. Data points with error bars are presented as mean ± s.d. derived from n = 3 independent experiments. (D) NIR-II bioluminescence intensity profiles on the dashed line in Fig. 2H (imaging window beyond 1000 nm).

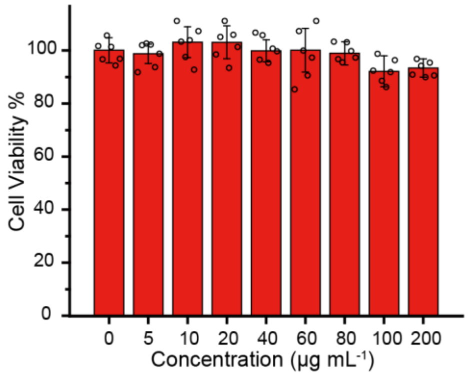

Supplementary Figure 10. *In vitro* viability values of CAOV-3 cells incubated with NIR-II-BPs at different concentrations for 24 h at 37 °C. No significant cellular toxicity was observed. Bars are presented as mean ± s.d.. All experiment groups were repeated for six times. Source data are provided as a Source Data file.

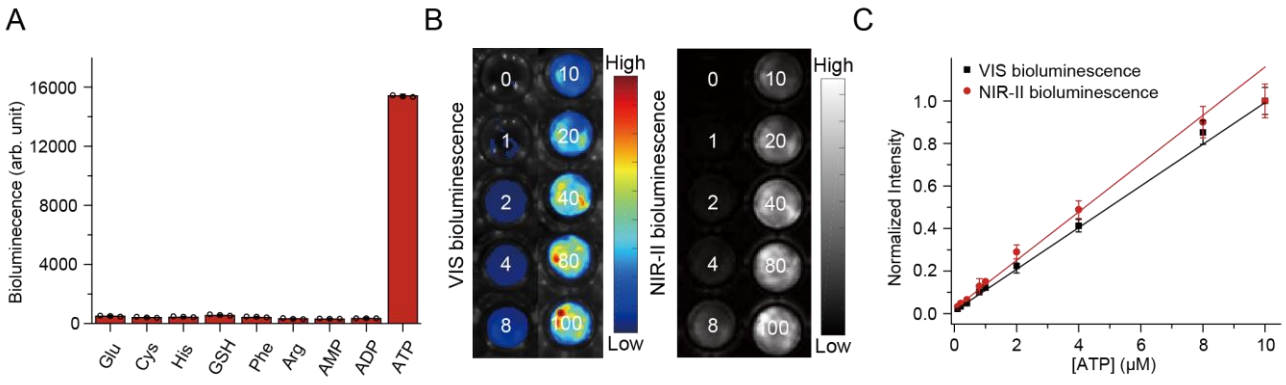

Supplementary Figure 11. *In vitro* ATP analysis with NIR-II-BPs. (A) The NIR-**II** bioluminescence of 10 μg mL^{-1} NIR-II-BPs following incubation with 2.0 mM ATP and other typical potential interfering species including Glutamine (Glu), Cysteine (Cys), Histidine (His), glutathione (GSH), Phenylalanine (Phe), Arginine (Arg), adenosine monophosphate (AMP), and adenosine diphosphate (ADP) in PBS buffer. Bars are presented as mean ± s.d. derived from n = 3 independent experiments. (B) VIS and NIR-II imaging of ATP assay and NIR-II-BPs incubated with 0, 1, 2, 4, 8, 10, 20, 40, 80, and 100 μM of ATP. (C) The relationship corresponding to visible bioluminescence of ATP assay (black) and NIR-II bioluminescence of NIR-II-BPs (red) verse the concentration of ATP ranging from 0 to 10 μM. Compared with the traditional ATP assay based on luciferase, the results acquired from NIR-II bioluminescence showed similar reaction activity but more sensitivity, with the limit of detection (LOD) at ~0.2 μM (SNR = 3), which was comparable to other reported methods.[9] Data points with error bars are present as mean ± s.d. derived from n = 3 independent experiments. Source data underlying **A** and **C** are provided as a Source Data file.

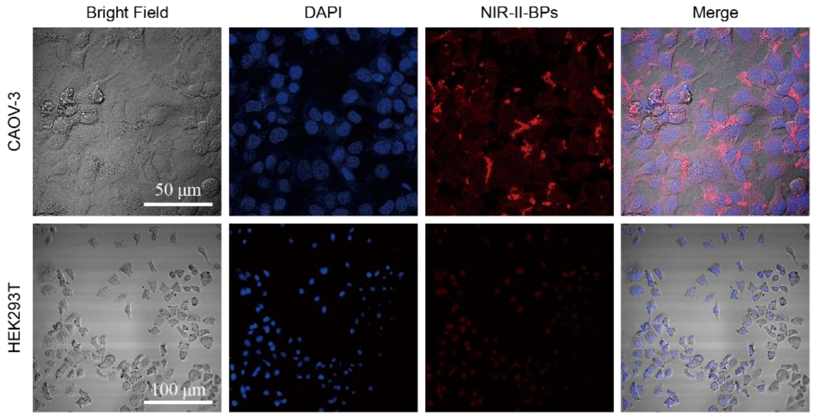

Supplementary Figure 12. Confocal microscopy images of CAOV-3 cells and HEK293T cells incubated with the NIR-II-BPs. Cell nuclei were stained with commercial DAPI for clarity. Human ovarian carcinoma cell CAOV-3 and human embryonic kidney cell HEK293T were chosen as the tumor and normal tissue models, respectively. After incubating with both cell lines, NIR-II-BPs could be successfully endocytosed. Repeated for 3 times in independent experiments.

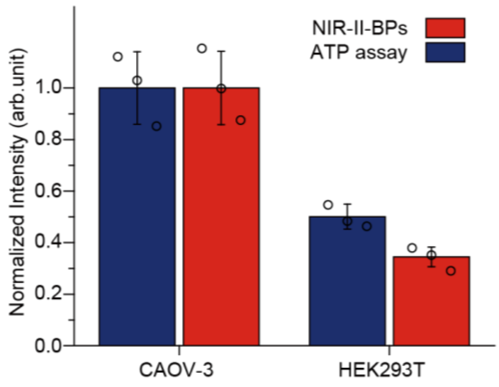

Supplementary Figure 13. *In vitro* metabolic states in CAOV-3 cells and HEK293T cells with NIR-II bioluminescence and conventional ATP assay. The results of our probes are consistent with those of commercial ATP assay and are comparable to the intracellular ATP concentrations reported in the literature.[10] Bars are presented as mean ± s.d. derived from n = 3 independent experiments. Source data are provided as a Source Data file.

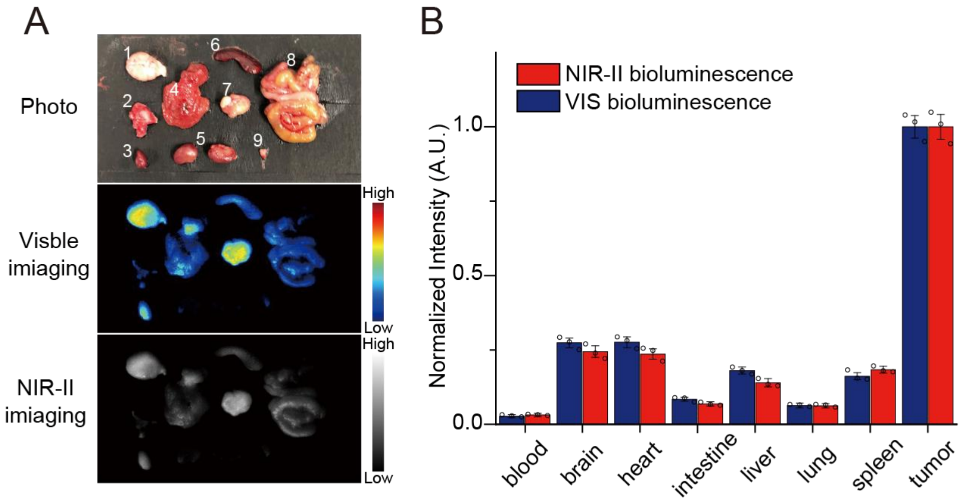

Supplementary Figure 14. *Ex vivo* metabolic states in different organs in a mouse. (A) The bright field, VIS bioluminescence imaging based on commercial ATP assay and NIR-II bioluminescence imaging based on the NIR-II-BPs of different organs. Nos.1-9 refers to brain, lung, heart, liver, kidneys, spleen, tumor, intestine and bladder. (B) Normalized intensity of different organ homogenates with NIR-II bioluminescence and conventional ATP assay, demonstrating excellent correlation between the two methods. The results were comparable with the reported ATP concentration in various organs.[10] Bars are presented as mean ± s.d. derived from n = 3 independent measurements. Source data are provided as a Source Data file.

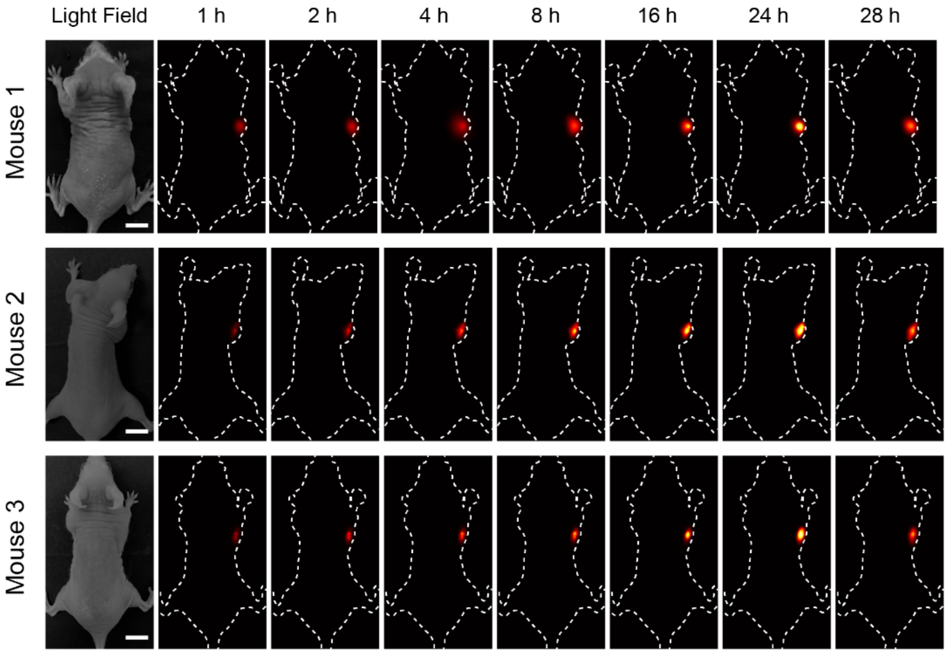

Supplementary Figure 15. NIR-II bioluminescence imaging results of xenografted tumor bearing mice (n=3) after tail injection with NIR-II-BPs for various hours. Scale bar, 1 cm.

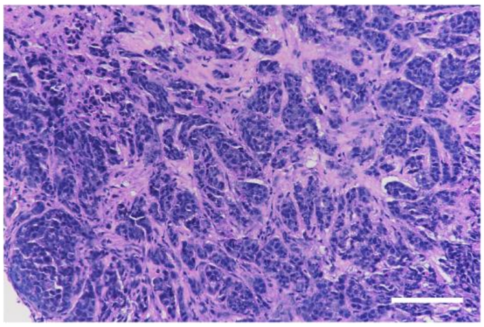

Supplementary Figure 16. H&E staining results of xenografted tumor in Supplementary Figure 15. The tumor tissue was confirmed to be malignant. Scale bars, 0.1 mm. Repeated for 3 times in independent experiments.

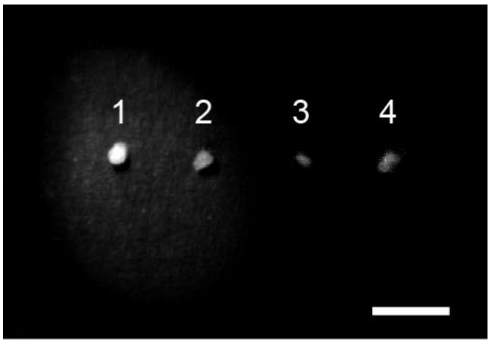

Supplementary Figure 17. NIR-II fluorescence image (1, 2) and NIR-II bioluminescence image (3, 4) of POM (1, 4) and SCM (2, 3) in Fig. 4F. Scale bar, 1 cm.

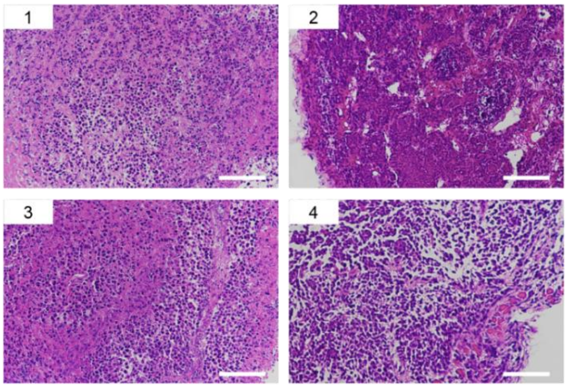

Supplementary Figure 19. H&E staining results of popliteal and sacral lymph nodes metastatic (No. 1-4) in Supplementary Figure 17. All the metastases were confirmed to be malignant. Scale bars, 0.1 mm. Repeated for 3 times in independent experiments.

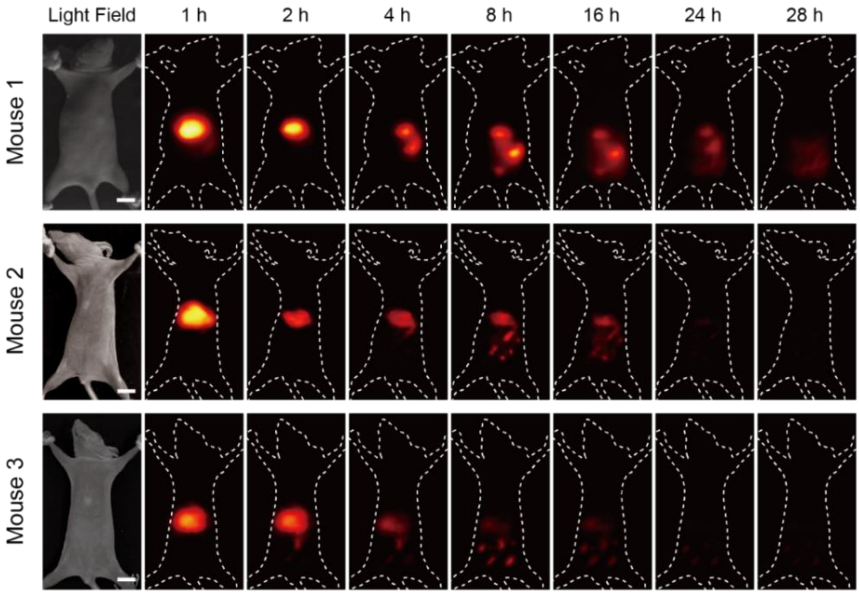

Supplementary Figure 19. NIR-II bioluminescence imaging results of peritoneal metastases bearing mice (n=3) after tail injection with NIR-II-BPs for various hours. Scale bar, 1 cm.

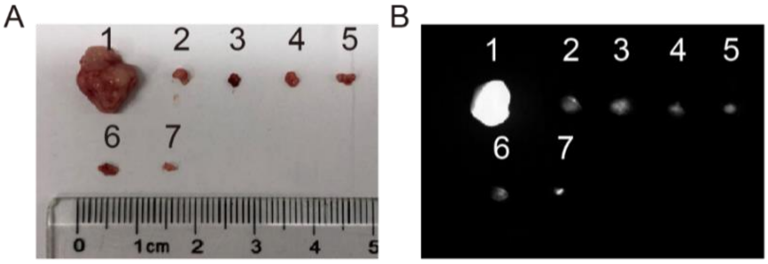

Supplementary Figure 20. Optical image (A) and the NIR-II bioluminescence image (B) of resected tumors in Fig. 6F.

Supplementary Tables

Supplementary Table 1. Photophysical properties of FD-1029 in various solvent and micells.

Solvents	λ_{abs}[a]	λ_{em} (nm)[b]	Stokes shift (nm)	$\varepsilon_{max}\times10^5$ ($cm^{-1}mol\cdot L^{-1}$)	ϕ_r(%)[c]
CH_3OH	975	1008	33	1.28	0.882
CH_3CH_2OH	981	1022	41	1.86	0.567
DMSO	1000	1025	25	1.06	0.120
PBS	985	1029	44	0.62	0.032
Micelles	977	1029	52	0.20	0.029

[a] The maximal absorption wavelength of FD-1029. [b] The maximal emission wavelength of FD-1029. [c] The relative fluorescence quantum yield of FD-1029 in different solvent and micelles by using IR-26 in 1,2-dichloroethane as a reference system (quantum yield ~ 0.05%).[11]

Supplementary Table 2. The concentration of NIR-II-BPs compositions.

NIR-II-BPs compositions	Concentration ($nmol\cdot mL^{-1}$)
Micelles[a]	0.36 ± 0.02
DSPE-PEG2000[b]	32.4 ± 1.8
Cy5[c]	0.95 ± 0.01
Cy7.5[c]	2.14 ± 0.10
FD-1029[c]	9.15 ± 0.22
Luciferase[d]	1.76 ± 0.12

[a] The micelle concentration is estimated by the concentration of DSPE-PEG2000 taking account an aggregation number near 90.[12] [b] The concentration of DSPE-PEG2000 is determined by colorimetric method (Supplementary Figure 5A). [c] The concentration of organic dyes are determined by spectrophotometric method (Supplementary Figures. 5C-E). [d] The concentration of luciferase is determined by Bradford assay (Supplementary Figure 5B). The ratio of Luciferase to the micelles was ~5.

The concentration of DSPE-PEG2000 in NIR-II-BPs was determined to be 90.9 ± 5.0 μg mL^{-1} through a colorimetric method (Supplementary Figure 5A), in which DSPE-PEG2000 reacted with barium chloride and iodine to form a barium iodine complex compound. Considering the molar mass of DSPE-PEG2000 was 2805.5 g mol^{-1}, the molar concentration of DSPE-PEG2000 in NIR-II-BPs was 32.4 ± 1.8 nmol mL^{-1}. Then, the micelle concentration was estimated to be 0.36 ± 0.02 nmol mL^{-1} with the concentration of DSPE-PEG2000 by taking account an aggregation number of 90. The concentration of luciferase protein was determined to be 0.109 ± 0.07 mg mL^{-1} with Bradford assay (Supplementary Figure 5B) Considering the molar mass of luciferase is ~62 KDa, the molar concentration of luciferase conjugated on NIR-II-BPs was 1.76 ± 0.12 nmol mL^{-1}. Hence, the ratio of luciferase to micelles is calculated to be ~5 (1.76/0.36 = 4.9).

Supplementary References

1. Williams, A. T. R., Winfield, S. A., Miller, J. N. Relative fluorescence quantum yields using a computer-controlled luminescence spectrometer. *The Analyst* **108**, (1983).
2. Clegg, R. M. Chapter 1 Förster resonance energy transfer—FRET what is it, why do it, and how it's done. In: *Fret and Flim Techniques*) (2009).
3. So, M. K., Loening, A. M., Gambhir, S. S., Rao, J. Creating self-illuminating quantum dot conjugates. *Nat. Protoc.* **1**, 1160-1164 (2006).
4. Li, B. H., Lu, L. F., Zhao, M. Y., Lei, Z. H., Zhang, F. An Efficient 1064 nm NIR-II Excitation Fluorescent Molecular Dye for Deep-Tissue High-Resolution Dynamic Bioimaging. *Angew. Chem. Int. Edit.* **57**, 7483-7487 (2018).
5. Sun, C.*, et al.* J-Aggregates of Cyanine Dye for NIR-II in Vivo Dynamic Vascular Imaging beyond 1500 nm. *J. Am. Chem. Soc.* **141**, 19221-19225 (2019).
6. Wu, M., Kusukawa, N. SDS agarose gels for analysis of proteins. *BioTechniques* **24**, 676-678 (1998).
7. Chung, T.-W., Chung, C.-H., Lue, Y.-F. A Colorimetric Method for Determining Distearoylphosphatidylethanolamine– Polyethylene Glycol 2000 in Blood Suspension. *Anal. Biochem.* **285**, 264-267 (2000).
8. Kruger, N. J. The Bradford method for protein quantitation. *Methods Mol. Biol.* **32**, 9-15 (1994).
9. Shen, Y. Z., Tian, Q., Sun, Y. D., Xu, J. J., Ye, D. J., Chen, H. Y. ATP-Activatable Photosensitizer Enables Dual Fluorescence Imaging and Targeted Photodynamic Therapy of Tumor. *Anal. Chem.* **89**, 13610-13617 (2017).
10. Traut, T. W. Physiological Concentrations of Purines and Pyrimidines. *Mol. Cell. Biochem.* **140**, 1-22 (1994).
11. Brown, C. M., Reilly, A., Cole, R. W. A Quantitative Measure of Field Illumination. *Journal of Biomolecular Techniques : JBT* **26**, 37-44 (2015).
12. Kastantin, M., Ananthanarayanan, B., Karmali, P., Ruoslahti, E., Tirrell, M. Effect of the Lipid Chain Melting Transition on the Stability of DSPE-PEG(2000) Micelles. *Langmuir* **25**, 7279-7286 (2009).
