# Supplementary material for: NIR-II bioluminescence for in vivo high contrast imaging and in situ ATP-mediated metastases tracing
Source: Nat Commun. 2020 Aug 21;11:4192. doi: 10.1038/s41467-020-18051-1 (PMC7442788; doi:10.1038/s41467-020-18051-1)
Supplement: Supplementary file 3 — Reporting Summary [file 41467_2020_18051_MOESM3_ESM.pdf]

nature research

Corresponding author(s): Fan Zhang
Last updated by author(s): Jul 1, 2020

Reporting Summary

Nature Research wishes to improve the reproducibility of the work that we publish. This form provides structure for consistency and transparency in reporting. For further information on Nature Research policies, see Authors & Referees and the Editorial Policy Checklist .

Statistics

For all statistical analyses, confirm that the following items are present in the figure legend, table legend, main text, or Methods section.

n/a	Confirmed	
[ ]	[x]	The exact sample size (*n*) for each experimental group/condition, given as a discrete number and unit of measurement
[ ]	[x]	A statement on whether measurements were taken from distinct samples or whether the same sample was measured repeatedly
[x]	[ ]	The statistical test(s) used AND whether they are one- or two-sided<br>*Only common tests should be described solely by name; describe more complex techniques in the Methods section.*
[x]	[ ]	A description of all covariates tested
[x]	[ ]	A description of any assumptions or corrections, such as tests of normality and adjustment for multiple comparisons
[ ]	[x]	A full description of the statistical parameters including central tendency (e.g. means) or other basic estimates (e.g. regression coefficient) AND variation (e.g. standard deviation) or associated estimates of uncertainty (e.g. confidence intervals)
[x]	[ ]	For null hypothesis testing, the test statistic (e.g. *F*, *t*, *r*) with confidence intervals, effect sizes, degrees of freedom and *P* value noted<br>*Give P values as exact values whenever suitable.*
[x]	[ ]	For Bayesian analysis, information on the choice of priors and Markov chain Monte Carlo settings
[x]	[ ]	For hierarchical and complex designs, identification of the appropriate level for tests and full reporting of outcomes
[x]	[ ]	Estimates of effect sizes (e.g. Cohen's *d*, Pearson's *r*), indicating how they were calculated

Our web collection on statistics for biologists contains articles on many of the points above.

Software and code

Policy information about availability of computer code

Data collection	NIR-II Images were acquired with Lightfield 6.11 software. Thermal images were acquired with FLIR ResearchIR 2.0.
Data analysis	Statistical analyses were performed using Originlab 2017 and Microsoft Excel 2019. Images were processed with Matlab R2016a. Ball-and-stick models were performed with ChemDraw3D 18.0.

For manuscripts utilizing custom algorithms or software that are central to the research but not yet described in published literature, software must be made available to editors/reviewers. We strongly encourage code deposition in a community repository (e.g. GitHub). See the Nature Research guidelines for submitting code & software for further information.

Data

Policy information about availability of data

All manuscripts must include a data availability statement. This statement should provide the following information, where applicable:

- Accession codes, unique identifiers, or web links for publicly available datasets
- A list of figures that have associated raw data
- A description of any restrictions on data availability

The source data underlying Figs. 1-4 are provided as a Source Data file. Data supporting the findings of this study are available within the article and the associated Supplementary Information Section. Any other data are available from the corresponding authors upon reasonable request. Source Data are provided with this paper.

Field-specific reporting

Please select the one below that is the best fit for your research. If you are not sure, read the appropriate sections before making your selection.

Life sciences study design

All studies must disclose on these points even when the disclosure is negative.

Sample size	No sample-size calculation was performed. Animal sample size (n) was determined by the number of biological replicates necessary for ensuring statistical significance. The number of biological replicates are also clearly reported in the the relevant figure legends. For experiments other than animal studies, the sample size (n) of each experiment is provided in the corresponding figure captions in the paper and supplementary information. Sample sizes were chosen to support meaningful conclusions.
Data exclusions	No data were excluded from the analyses.
Replication	Animal experiments were performed on biological replicates following identical procedures to verify the reproducibility of the experimental findings. Other experiments were successfully repeated independently in triplicates (preparation and characterization of probes; in vivo imaging experiments).
Randomization	All samples/organisms were randomly allocated into experimental groups.
Blinding	Investigators were not blinded to group allocation during data collection and analysis, due to limited personnel.

Reporting for specific materials, systems and methods

We require information from authors about some types of materials, experimental systems and methods used in many studies. Here, indicate whether each material, system or method listed is relevant to your study. If you are not sure if a list item applies to your research, read the appropriate section before selecting a response.

Materials & experimental systems

n/a	Involved in the study	
☒	☐	Antibodies
☐	☒	Eukaryotic cell lines
☒	☐	Palaeontology
☐	☒	Animals and other organisms
☒	☐	Human research participants
☒	☐	Clinical data

Methods

n/a	Involved in the study	
☒	☐	ChIP-seq
☒	☐	Flow cytometry
☒	☐	MRI-based neuroimaging

Eukaryotic cell lines

Policy information about cell lines

Cell line source(s)	CAOV-3 cells, HEK293T cells and CT-26 cells were obtained from American Type Culture Collection (ATCC, Manassas, VA, USA)
Authentication	All cell lines were authenticated by Short Tandem Repeat test.
Mycoplasma contamination	All cell lines tested negative for mycoplasma contamination.
Commonly misidentified lines (See ICLAC register)	No commonly misidentified cell lines were used in the study.

Animals and other organisms

Policy information about studies involving animals; ARRIVE guidelines recommended for reporting animal research

Laboratory animals	Female BALB/c nude mice (5 weeks old, ~15 g)were obtained from Shanghai SLRC Laboratory Animal Centre (China). Mice were housing in independent ventilation cage (IVC) system under specific pathogen free (SPF) condition. Housing temperature was 22-25 degree Celsius, humidity was 35-45 %. The dark/light cycle was 12 h light/12 h dark.
Wild animals	The study did not involve wild animals.
Field-collected samples	The study did not involve sampled collected from the field.
Ethics oversight	All animal experiments were performed under the approval of Fudan University's Administrative Panel on Laboratory Animal Care.

Note that full information on the approval of the study protocol must also be provided in the manuscript.
